# Physical Activity and Nutritional Influence on Immune Function: An Important Strategy to Improve Immunity and Health Status

**DOI:** 10.3389/fphys.2021.751374

**Published:** 2021-10-08

**Authors:** Tianyi Shao, Henu Kumar Verma, Babita Pande, Vincenzo Costanzo, Weibing Ye, Yuyan Cai, L. V. K. S. Bhaskar

**Affiliations:** ^1^College of Teacher Education, Zhejiang Normal University, Jinhua, China; ^2^Department of Immunopathology, Institute of lungs Biology and Disease, Comprehensive Pneumology Center, Helmholtz Zentrum, Munich, Germany; ^3^Department of Physiology, All India Institute of Medical Science, Raipur, India; ^4^Department of Experimental, Diagnostic and Specialty Medicine, University of Bologna, Bologna, Italy; ^5^College of Physical Education and Health Sciences, Zhejiang Normal University, Jinhua, China; ^6^Department of Physical Education, Guangdong University of Technology, Guangzhou, China; ^7^Department of Zoology, Guru Ghasidas Vishwavidyalaya, Bilaspur, India

**Keywords:** nutrition, physical activity, immune fuction, exercise, aging

## Abstract

Physical activity (PA) and nutrition are the essential components of a healthy lifestyle, as they can influence energy balance, promote functional ability of various systems and improve immunity. Infections and their associated symptoms are the common and frequent challenges to human health that are causing severe economic and social consequences around the world. During aging, human immune system undergoes dramatic aging-related changes/dysfunctions known as immunosenescence. Clinically, immunosenescence refers to the gradual deterioration of immune system that increases exposure to infections, and reduces vaccine efficacy. Such phenomenon is linked to impaired immune responses that lead to dysfunction of multiple organs, while lack of physical activity, progressive loss of muscle mass, and concomitant decline in muscle strength facilitate immunosenescence and inflammation. In the present review, we have discussed the role of nutrition and PA, which can boost the immune system alone and synergistically. Evidence suggests that long-term PA is beneficial in improving immune system and preventing various infections. We have further discussed several nutritional strategies for improving the immune system. Unfortunately, the available evidence shows conflicting results. In terms of interaction with food intake, PA does not tend to increase energy intake during a short time course. However, overcoming nutritional deficiencies appears to be the most practical recommendation. Through the balanced nutritious diet intake one can fulfill the bodily requirement of optimal nutrition that significantly impacts the immune system. Supplementation of a single nutrient as food is generally not advisable. Rather incorporating various fruits and vegetables, whole grains, proteins and probiotics may ensure adequate nutrient intake. Therefore, multi-nutrient supplements may benefit people having deficiency in spite of sufficient diet. Along with PA, supplementation of probiotics, bovine colostrum, plant-derived products and functional foods may provide additional benefits in improving the immune system.

## Introduction

It is well established that physical activity (PA) and healthy nutrition are vital lifestyle factors that influence lifelong health by improving body composition, musculoskeletal health, physical and cognitive performance. Optimal physical activity and proper nutrition are also important to prevent metabolic diseases such as obesity, diabetes mellitus, and cardiovascular disease. The WHO has identified lack of physical activity as a significant risk factor for global mortality. They recommended that some amount of physical activity is better than none and more physical activity is better for optimal health outcomes (Reardon et al., 2019; Bull et al., 2020). While the health benefits of nutrition and physical activity are frequently studied separately, it is now becoming increasingly clear that combining nutrition and physical activity can produce more significant positive health consequences and boost the immune system when compared to strategies that focus solely on one or the other. Regular moderate exercise has been shown to reduce the risk of infection compared to a sedentary lifestyle (Silişteanu and Covaşă, 2015). Similarly, a large systematic analysis from 195 countries shows that a poor diet lacking optimal nutrition is linked to adverse health outcomes. While association among many lifestyle (physical activity and diet)-related benefits, such as improved fitness, overall health/well-being, and perceived quality of life, are well documented (GBD 2017 Diet Collaborators, 2019).

The potential benefits to immunity and infection susceptibility are significant but widely ignored. Infection, including respiratory tract infection and its associated symptoms, is the most common and frequent presentation, and it can have severe economic and social consequences (Varricchio et al., 2020). It has also been noticed that prolonged periods of exercise and intense training are linked to an increased risk of infection. Acute bouts of strenuous exercise cause a temporary dwindle in the immune function that can last up to 24h after the workout (Walsh et al., 2011). During an individual lifespan, the PA or the nutritional requirement alters during different development stages and naturally decreasing with aging. Age-related quantitative and qualitative changes occur in the immune system known as immunosenescence (Longo et al., 2015).

**Figure 1:**
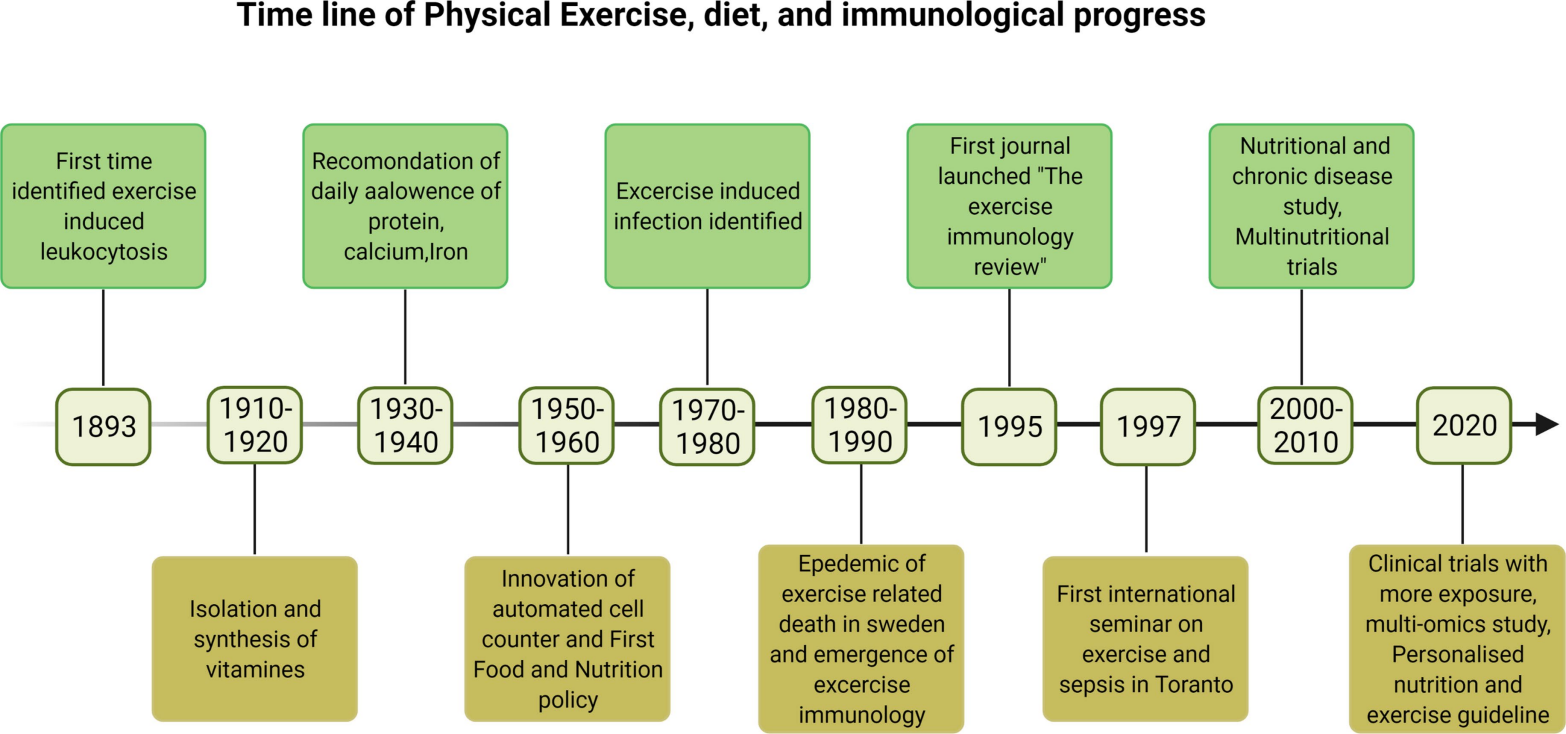
The timeline of the early development of immunology and discoveries in physical exercise and diet impacting the immune system (Doherty and Robertson, 2004; Kaufmann, 2019).

Clinically, immunosenescence refers to the gradual deterioration of immune system mostly observed with aging of an individual that increased the exposure to infections, increased viral reactivation, and reduced vaccine efficacy and is linked to impaired immune responses leading to pathologies (Weyand and Goronzy, 2016). Therefore majority of older adults are more prone to death by suffering from multiple chronic diseases, and they are living lives in poor health and with disabilities due to the burden of chronic diseases. This is expected to put a strain on healthcare and social costs. Lack of physical activity, progressive loss of muscle mass, and the concomitant decline in muscle strength facilitate immunosenescence and inflammation (Fulop et al., 2020). The early history of immunological changes with physical activity and importance of nutrients is poorly documented. Some recent findings on this context are reported in timeline Figure 1. Although research into the effects of physical activity and exercise on the immune system is ongoing, evidence suggests that long-term PA activity is beneficial for immune enhancement and infection prevention. In this review, we have discussed the role of PA and nutrition in boosting the immune system. We have also discussed PA and exercise, and their relationship to various nutritional strategies for improving the immune system.

## Overview of the Immune System

The immune system is a complex system, and it is influenced by an ideal environment without nutrient deficiencies that are critical in immune cell triggering, interaction, differentiation, or functional expression (Zheng et al., 2020). There are two types of the immune response. Innate immunity is the first immune response that comes into play in response to invading pathogens/foreign bodies and is quick but not specific. The components of the innate immune responses are phagocytes like macrophages and monocytes, neutrophils, mast cells, eosinophils, etc. The more specific and long-term immune response is the adaptive immune response. T and B cells are the specialized cells for long-term immunity. These cells recognize the antigen elicit specific immune responses rapidly against particular pathogens. The cytotoxic T cells with CD8 receptor kill the infected cells or tumor cells, and the T helper cells with CD4 receptor help other immune cells. B cells produce different antibody or immunoglobulin (Ig) classes that target antigens and destroy the pathogens or any foreign antigens. The severity of infection varies by gender and age, and it is linked with comorbidities; as a result, the immune system develops. Several factors influence the immune system and its competence, including nutrition (Maggini et al., 2018).

## Relationship Between Physical Activity and Immune System

The lack of sufficient daily PA is an underappreciated primary cause of the majority of chronic conditions. Overwhelming evidence supports the notion that decreases in daily PA are the primary causes of chronic diseases and rehabilitative treatment for inactivity-caused dysfunctions (Booth et al., 2017). Although the terms “physical activity” and “exercise” are frequently used interchangeably, it is essential to understand the differences. According to the Center for disease control (CDC), PA is any bodily movement produced by the contraction of skeletal muscle that increases energy expenditure at baseline level. Exercise is a type of physical activity that is planned and carried out with the goal of improving physical fitness (Seibert et al., 2019).

Majority of research conducted over the past century has investigated how physical activity affects the immune system (Campbell and Turner, 2018; Chastin et al., 2021). Recently two meta-analyses have shown that physical activity prevents the risk of upper respiratory tract infections and provides immunity, nevertheless supportive data are minimal and the results are inconclusive due to a small number of samples and unavailability of published studies (Rocco et al., 2018; Grande et al., 2020). According to the meta-analyses, regular physical activity is associated with 31% lower risk of infectious disease and 37% lower risk of infectious disease-related mortality.

Conversely, evidence is mounting that physical inactivity and its consequences, such as adipose tissue accumulation and muscle dysfunction, have a negative impact on both innate and adaptive immunity. Further, it is now widely accepted that intensive physical activity improves immune function and that once the recovery process is complete, beneficial adaptations occur. Physical activity interventions of 3–5 times per week for an average of 30min resulted in higher CD4 T cells and salivary immunoglobulin IgA and lower levels of neutrophils. Neutrophils are the most abundant white blood cells, the primary effectors of pathogen clearance, and the first white blood cells recruited during infection.

Furthermore, IgA has several other functions; the most crucial role is to act as an anti-inflammatory response and strengthen the mucosal barrier to pathogens and the body’s first line of defense (Hansen et al., 2019). They not only enhance the response and memory of other immune cells, but they are also involved in direct effector mediating pathogen clearance. The increased concentrations of CD4 T cells found in these analyses suggest that regular physical activity strengthens the immune system and results in a faster response (Swain et al., 2012; Laidlaw et al., 2016). Jung et al. (2018) found that physical inactivity and metabolic abnormalities are associated with reduced NK cell activity.

Neutrophils, in particular, play a significant role in chronic inflammation, and an elevated neutrophil count is frequently considered as a marker of chronic inflammation. A 10years follow-up study by Hamer et al. (2012) demonstrated that physically active participants at baseline contain lower C-reactive protein and interleukin-6 levels. Thus PA may be necessary for preventing the proinflammatory state with aging. Recently Fedewa et al. (2017) found that extensive exercise training is associated with decreased CRP levels.

This mounting evidence suggests that moderate to vigorous physical activity on a regular basis may play an essential role in immune system and response effectiveness, providing enhanced protective immunity against infections. Furthermore, more research in relation to stress and recovery responses and exercise is needed to understand the beneficial effects of exercise on immune functions. This implies that people should be encouraged to engage in daily PA in order to strengthen their immune system and reduce the risk of infectious disease and infectious disease-related mortality.

## Aging and the Immune System

The immune system represents a defense mechanism to protect the human body against foreign hosts, including bacteria and viruses, and alter intrinsic matter, such as cancer cells (Nicholson, 2016). The bone marrow is the main actor involved in the immune response. It contains hematopoietic cells responsible for the used production of immune cells, which migrate to peripheral lymphoid organs to complete their maturation and obtain the capacity to recognize non-self-components (Morsink et al., 2020).

Innate immunity refers to a non-specific and fast mechanism that can detect and destroy common microbial components. However, it is limited in the number of receptors to recognize specific pathogens. The innate response mainly includes the anatomical and physiological barriers, such as skin and low gastric pH, and the phagocytosis mechanism, mediated by a wide range of cells, including macrophages and natural killers (NK). Conversely, adaptive immunity constitutes a more complex response, typically mediated by T and B cells, which express a multitude of receptors to permit antigen identification. This type of immunity is highly specific and requires a migration of immune cells to infection sites to enter in contact with exogenous pathogens (Zhang et al., 2014).

After naive B and T cells are formed in the bone marrow and thymus, they migrate to secondary lymphoid tissues such as the spleen. This process is robust in children to generate a diverse immune repertoire and fill peripheral lymphoid compartments. Although aging decreases the number of naive B and T cells migrating from primary to secondary lymphoid organs, B and T cell development does not stop completely, even in the elderly, some functional thymic tissue exists (Nikolich-Zugich et al., 2012; Montecino-Rodriguez et al., 2013). As we all know, an increased number of memory T cells is a well-known feature of aging. When these cells are re-exposed to the specific invading antigen, they quickly divide and express molecules such as cytokine proteins supporting the fight against infection. When the pathogens are eliminated, most effector cells die, but a small pool of protective memory cells remains, prepared to respond rapidly if reinfection occurs (Aiello et al., 2019). This phenomenon is a significant factor contributing to the accumulation of CD8^+^ memory cells in people aged 60years and older (Kaech and Cui, 2012).

**Figure 2:**
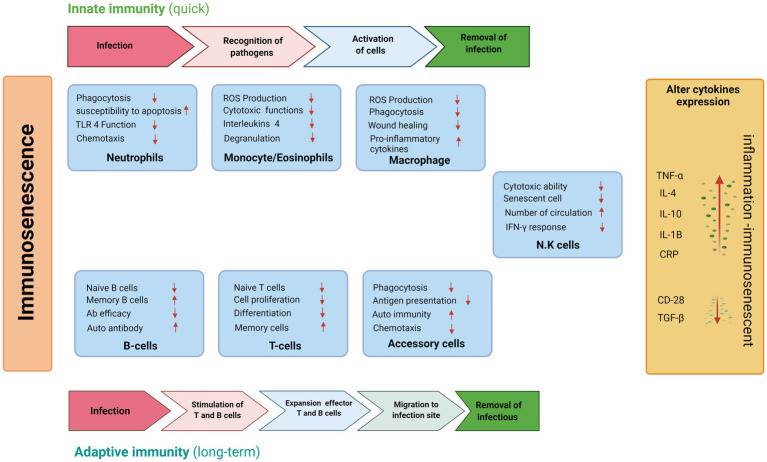
Schematic representations of age-related changes in the innate and adaptive immune system during aging in inactive individuals.

Similarly, aging has a significant impact on B cell functions, as many other B-cell biomarkers of aging have been identified. Natural aging is accompanied by progressive biological changes in the immune system (Figure 2). They are linked to low recombination and somatic hypermutation of immunoglobulin genes, resulting in decreased antibody production and decreased vaccination or infection. These mechanisms are responsible for changes that occur with age in both innate and adaptive immunity. They may be responsible for autoimmune disorders, cancer, and an inadequate response to vaccination (Blomberg and Frasca, 2013).

Macrophages are among the first cells to react when exogenous materials infringe the primary human protective barriers. They accomplish many crucial functions to destroy the microbes, including triggering inflammation and phagocytosis. Furthermore, macrophages are also involved in tissue homeostasis and wound healing through the recruitment of fibrogenic factors and the elimination of cellular debris (Zhang et al., 2014). It is widely accepted that aged macrophages exhibit a lower expression of major histocompatibility complex II (MHC-II) and toll-like receptors (TLR; Handunnetthi et al., 2010), which may impair the antigen presentation mechanism and reduceds the production of interleukin (IL)-7 (Mittal and Roche, 2015). In addition, old macrophages are associated with reduced phagocytosis and chemotaxis events as well as increased levels of proinflammatory cytokines, including tumor necrosis factor α (TNFα), IL-6 and IL-1β (Turner et al., 2014; Kany et al., 2019).

Indeed, an imbalanced cytokine expression represents a hallmark of the aging process, as confirmed by an overall increase in c-reactive protein (CRP), impairment of transforming growth factor-beta (TGF β) pathway and other cytokines, such as IL-4 and IL-10 (Ferrucci and Fabbri, 2018; Kong et al., 2020). This contributes to a significant age-related reduction in inflammation, leading to a higher risk of mortality in the elderly population (Batista et al., 2020). Aging caused a decrease the production of IL-12, a fundamental interleukin required for T-cell immune response, decreases, resulting in an impairment of the antigen presentation process mediated by accessory cells such as dendritic and langerhans cells (Mbongue et al., 2014).

Another example of aging-related immune system impairment is the altered function of neutrophils, which are the main characters in the fight against microbial infection. However, there is no reduction in their number during chemotaxis, phagocytosis, and superoxide production, which leads to increased susceptibility to cell death by apoptosis (Ponnappan and Ponnappan, 2011). Mathur et al. (2008) showed some age-related functional differences by carrying out *in vitro* experiments on eosinophils collected by peripheral blood of young and old patients. Remarkably, eosinophilic cells of aged people exhibited a substantial decline in the degranulation process and a slight reduction in ROS production, which suggest an alteration of cytotoxic functions.

NK cells are leucocytes designed to destroy tumor or virus-infected cells by releasing lytic proteins such as granzymes and perforin. Older people have higher NK cells, as evidenced by more elevated specific markers, particularly CD56, compared to the young (Paul and Lal, 2017).

People regularly involved in physical activity show lower signs of immunosenescence. Recent studies highlighted a higher frequency of naïve T-cells and B regulatory cells as well as increased levels of thymoprotective cytokines in elder people regularly involved in cycling activity during their lives compared to untrained people (Duggal et al., 2018; Tylutka et al., 2021). Moreover, influenza vaccine responses are improved in elder trained population, as demonstrated by higher antibody titers against H1N1 and H3N2 strains of influenza A following 10months of aerobic physical exercise (Simpson et al., 2015). In addition, old trained people exhibit a lower extent of inflammatory markers compared to inactive people. This could be at least partially due to the action of muscle mass, which have immunoprotective and anti-inflammatory effects by releasing protective factors called “myokines” (Pedersen and Febbraio, 2012).

On the other hand, immune system is also affected by dietary lifestyle. There is evidence that lack or deficiencies of minerals and vitamins as well as an excess of saturated fatty acids negatively affect immune responses. An important difference in terms of immunity changes can be made among healthy and undernourished elder people. Pae et al. (2012) show that old people with protein dietary deficiency exhibit an alteration of most cell-mediated immunity parameters, such as lymphocytes proliferation and cytokines synthesis, as well as reduced macrophage function and phagocytosis.

Diminished nutritional status, including an abnormal value in serum albumin, zinc or folate levels, is associated with significant change in T-cell subset and in T-cell function (Raiten et al., 2015). Moreover, a study proved that supplementation with vitamins and micronutrients, such as zinc and selenium, improved immune response in old people, as justified by raise in CD4^+^ T-cells subset, NK cells, and allowed a better antibody response against influenza vaccines (Schmoranzer et al., 2009).

## The Role of Nutrition in the Immune System

Nutritional interventions have been a part of some cultures or traditions like the Indian and the Chinese for a long time. Thus, nutrition intervention could be an essential therapeutic way to manage many diseases in hospitals, clinics, and homes (Reber et al., 2019). Nutrition supplementations reduce not only chronic diseases but also immune-mediated side effects. Rather than looking at energy boost, adequate and proper nutrition provides much more by enhancing the immune functions and providing a healthier life.

The immune system is the body’s defense system that ensures overall health and survival; it protects the body from invading pathogens or microflora residing in the body (gut microbiome) and regulating its inner system. To combat the infection, the immune system remains in active mode all the time. There is more energy expenditure for adequate clearance of the pathogens; thus, the body’s immune system needs a continuous energy supply. At this time, sufficient nutrition only can provide enough power to immune cells during the infection.

The invading pathogens lead to loss of appetite, malabsorption, increased nutrient demand, and loss of critical endogenous nutrients. The risk and severity of infection differ according to immune competence, development, maturation, and the immune system’s decline. Therefore, nutrition is one of the significant factors impacting the body’s immune system or defense mechanism.

Thus nutrition, primarily adequate and appropriate nutrition, is an essential determinant of optimal immune functions such as trigger of immune cells, interaction, differentiation, or functional expression of these cells. Malnutrition deteriorates the immune system and increases the vulnerability to infectious pathogens (Zheng et al., 2020). Nutrition can play a role in effective responses against pathogens and modulates the immune functions to combat stress, disease, and injury.

Nutrition can come from either an exogenous or endogenous source. Exogenous sources are diets that provide vitamins or micronutrients. Some micronutrients and dietary components play specific immune roles, such as activating immune functions. Endogenous sources, such as the stored form of energy in body cells or tissues, become instantaneous sources that can be derived through various mechanisms, such as when required, in the absence or insufficiency of exogenous nutrients.

## Carbohydrate

The primary energy source is carbohydrate in the human body, and it can also be found in immune cell components. It has been reported that carbohydrates can help boost the immune system. It has been proven that carbohydrates can modulate the immune system properties as studied in exercise or athlete performance (Gunzer et al., 2012; Kerksick et al., 2018). Individuals with a poor carbohydrate diet are more prone to immune dysfunction during exercise; however, it is also helpful during exercise-induced adaption like mitochondrial biogenesis and lipolysis (Mata et al., 2019).

After exercise, a balanced and simple carbohydrate diet, such as 30–60g/h, has immunomodulatory effects as observed in athletes. These effects are decreased systemic IL-6 release neutrophils, monocytes, lymphocytes, less suppression of CD4^+^ and CD8^+^ T cells, which causes leukocyte distribution and immune responses such as neutrophil degranulation and oxidative stress, lymphocyte proliferation and functions (Maydych et al., 2017; Peake et al., 2017).

However, the impact of carbohydrate supplementation to recover from upper respiratory tract infections (URTI) during strenuous exercise or games in athletes has been least explored (Nieman and Mitmesser, 2017). In this context, conflicting reports showed the ineffectiveness of carbohydrate supplementation to minimize URTI risk (Jones and Davison, 2019). While animal models have shown that a carbohydrate-rich diet reduces the risk of herpes simplex virus 1 [HSV-1] infection and mortality (Harper et al., 2021), it has also been shown that carbohydrate intake after exercise debilitates human T cells’ declining migration towards human rhinovirus–infected airway epithelial cells, as seen *in vitro* (Peake et al., 2017). Therefore, more research regarding the effectiveness of carbohydrate supplementation with clinical relevance in a case like URTI is required.

Further, it has been shown that the activity of NK cells increased by taking the carbohydrate-rich diet compared with the fat-rich diet during training (Gunzer et al., 2012; Clark and Mach, 2016). These researchers have documented that a fat-rich diet is harmful to the immune system compared to a carbohydrate-rich diet, though the reason is not clear.

### Carbohydrate Supplementation Before and During Exercise

Long-term carbohydrate supplementation during strenuous exercise has been shown to reduce exercise-induced circulating cytokines and support the redistribution of immune cells such as neutrophils, monocytes, NK cells, and lymphocytes (Nieman and Wentz, 2019). The immune-modulatory role of carbohydrates is more effective when there are optimal blood glucose concentrations and reduce stress hormones such as catecholamines and glucocorticoid secretion during and after exercise (Nieman and Pence, 2020). The carbohydrate intake during exercise did not change the muscle glycogen; however, the muscle glycogen has decreased in response to systemic release of IL-6 during exercise, reducing carbohydrate supplementation (Alghannam et al., 2018).

According to a study, carbohydrate consumption reduces antigen-stimulated proliferative lymphocyte responses on the second day before exercise while an increasing trend was observed in lymphocyte proliferative responses to mitogen stimulation after exercise (Bishop et al., 2005). Thus, the carbohydrate supplementation as a function of time (day) of pre and post-exercise has a crucial role in adaptive immune response and is helpful to enhance immune functions during long-term exercise or do two consecutive exercises with short recovery periods.

## Micronutrients

In the present scenario, micronutrients and immune function studies have much interest. Vitamin C (ascorbic acid or antioxidant vitamin), vitamin D, and zinc are all essential contributors to immune response. Immune dysfunction can also be caused by a lack of certain micronutrients, such as iron, zinc, and vitamin A.

It has been assumed that immune dysfunction caused by any micronutrient deficiency can be restored by consuming that individual micronutrient and adjusting to the optimal or normal level in the body (Gombart et al., 2020). The benefits and effects of micronutrients on immunity have been widely studied (Maggini et al., 2018). The deficiency of micronutrient zinc that acts as a cofactor in enzymes leads to immune dysfunction related to adaptive and innate immune response (Pecora et al., 2020). The role of zinc specifically has been found to improve patients’ immunity with sepsis (Alker and Haase, 2018). Evidence also shows that additional intake of certain micronutrients (more than current RDAs) improves the immune system and reduces susceptibility to infections (Berger et al., 2021).

### Antioxidant Vitamins

Vitamins E and C are the antioxidants that occur naturally in fruits and vegetables which protect the body from free oxygen radicals formed during inflammatory reaction or activation of phagocytosis of invading microorganisms (Kurutas, 2016). The recommended quantity of vitamin E is 15mg for adult females and males, and that of vitamin C is 75mg and 90mg for females and males, respectively; so there could be possible health hazards if the dose exceeds. (Ristow et al., 2009) reported that intake of a higher dose of vitamins C and E in combination after 4weeks of intense exercise prevents ROS defense-related enzymes in human skeletal muscle. Therefore, reports regarding the benefits of vitamins E and C administration alone or in combination on muscle mass and strength is also varying. Intense exercise generates free radicals that are the primary reason for muscle injury, fatigue, and poor performance (Apostolopoulos et al., 2015). This also indicates that athletes who are generally engaged in exercise seem to experience greater efficacy of intake of vitamins E and C and improvement in athletic performance when taken in adequate amount.

It has also been examined that vitamin E supplements are insufficient to improve the performances at altitude, specifically they are not effective for quick and short-term improvement in an athlete’s performance. Enhanced antioxidant capacity decreases the systemic inflammatory biomarkers in elite endurance athletes at altitude (Koivisto et al., 2019). The instances cited above signify that antioxidant supplements like vitamins E and C are more likely to hinder the anabolic signaling pathways that deteriorate the adaptations to resistance training (Dutra et al., 2019). Thus it is suggested that athletes should be aware while consuming antioxidants like vitamins E and C directly. Fruits and vegetables are good sources of vitamin E and C. These nutrients are not in concentrated form but present along with other minerals and bioactive compounds that meet the required amount of vitamins E and C.

As a water-soluble vitamin, vitamin C is considered safe and non-toxic with a few side effects, so many used to take a high dose of vitamin C supplements (Hemila and Chalker, 2013). The routine and regular consumption of vitamin C is very well known to reduce the chances of common cold and, if contracted, reduce the duration of common cold (Hemila and Chalker, 2013). Vitamin C also showed an inverse relationship with phagocytosis (Fantacone et al., 2020). There are cases where the increased antioxidant is beneficial; for example, high doses of antioxidant vitamins (10× RDA) have affectively minimized the chances of infection in ultra-endurance athletes (Salehi et al., 2018). In football players, reduction in oxidative stress has been documented when vitamins C and E were consumed in combination (De Oliveira et al., 2019). Administration of vitamin E alone before exercise under the hypoxic condition at high altitude restores the interleukin (IL)-6, TNF-α, IL-1ra and IL-10 to normal level (Santos et al., 2016). It has been suggested that it might be due to antioxidant like vitamin C mediated decreased level of stress hormone and cytokine production.

A high antioxidant content in body attenuated IL-6 responses to a stressor (exercise) through reducing IL-6 production in healthy individuals (Walsh, 2019). Reports also purported about the side effects of intake of high dose single type of antioxidant. A high doses of vitamin E potentially acts as modulator proinflammatory agents, oxidative stress and NF-κB pathway having role in neuroprotection and cancer (Ungurianu et al., 2021). However, a high dose of antioxidant seems to be favorable to individuals at high risk of URTI to enhance the immunity to reduce URTI risk (Davison et al., 2014). It has been alleged that the high concentration of these antioxidants interferes with the normal physiological functions and decreases benefits of physical activity or the normal training response such as decrease in muscle strength (Paulsen et al., 2014).

#### Vitamin D

Vitamin D is an essential nutritional supplement that strengthens the bones and is vital for calcium and bone homeostasis. Substantial evidence supports the role of low vitamin D content with compromised immunity and increased risk of illness. The role of vitamin D has been explored in immune function like deficiency of this vitamin has been linked to increased susceptibility to infection. An association between vitamin D content and Acute Respiratory Tract Infection risk has also been observed (Pham et al., 2019).

Vitamin D is a secosteroid hormone known to interact with more than 200 genes *via* nuclear receptors expressed on various tissues of the body (Nurminen et al., 2019), including the immune cells. Thus vitamin D facilitates the innate and adaptive immune responses and modulates a wide range of physiological functions. The immune cells such as B cells, T cells, and antigen-presenting cells expressed the receptor for the vitamin D and can synthesize active vitamin D metabolite. Vitamin D receptors are important to activate the cathelicidin antimicrobial peptide (CAMP) in activated monocytes or macrophages. Vitamin D participates in innate antimicrobial response through activation of Toll-like receptors (TLR) that increased the expression of both 1-α-hydroxylase and vitamin D receptors (VDR; Di Rosa et al., 2011). It inhibits B cell proliferation and blocks B cell differentiation and immunoglobulin secretion (Medrano et al., 2018). In addition, vitamin D acts in an autocrine manner to elicit an immune response. Deficiency in vitamin D has been shown to augment autoimmunity besides increasing the risk of infection. Vitamin D supplementation is thus helpful to individuals suffering from vitamin D deficiency mediated autoimmune diseases.

Vitamin D also has an inverse relationship with the reactive oxygen species production (Berridge, 2016). The majority of individuals from different countries, primarily from developed countries, suffer from vitamin D deficiency (Sizar et al., 2021). Though there are dietary sources for vitamin D, it is mainly synthesized beneath the skin when exposed to solar ultraviolet B radiation from sunlight. Thus seasonal availability of direct sunlight, for example, lack of solar light in winter, affects vitamin D synthesis, especially in temperate countries.

Accordingly, vitamin D deficiency can be adjusted with intake of vitamin D rich diet or exposure to sunlight. Once the normal level is achieved, there may be no further benefits from further vitamin D consumption. Studies to date are mostly cross-sectional or observational. Only the beneficial effect of vitamin D supplementation and deficiency surfaced the respective vitamin as an index of compromised immune function. Indeed vitamin D intervention based on more research could evaluate the actual impact of vitamin D deficiency or overdose.

### Multivitamin and Mineral Supplement

Studies show that a multivitamin and mineral supplement (MVM) improves individuals’ immune functions and immune status, as documented in older people with deficiency in minerals and vitamins. There is a natural decline in immune functions during aging, such as decrease in neutrophil function that amplifies by diet deficit in multiple vitamin and mineral lacks (Zhang et al., 2015a; Maggini et al., 2018).

A MVM supplement contains at least three vitamins and at least one mineral; therefore, combinations of nutrients (multivitamins and mineral supplements) could be a more effective means to recover from a deficiency than a single nutrient, which may imbalance the nutrient content in the body (Bird et al., 2017). Experimentally, a good supplement of MVM containing a high amount of many redox-active and immunomodulatory micronutrients such as Redoxon® Vita Immune (VI) MVM supplements has been studied. This product intake for at least 12weeks reduces the incidence of infection in older adults. It boosts serum vitamin C and zinc content compared to older adults without MVM supplements who were more susceptible to infections (Fantacone et al., 2020). However, the respective authors did not observe any significant changes in neutrophil function compared to a placebo group; this may account for the participants’ optimal blood and zinc content.

Additionally, the deficiency indicates physiological demand for normal immune functions or vitamins/minerals for enhancing athletic/training performance. Furthermore, nutrients obtained from food rather than from supplements could provide a natural blend of nutrients, accomplishing the goal of a multi-nutrient supplement. It may also provide nutrients that work synergistically and are more beneficial than single nutrients.

Supporting this, it has been observed that pregnant women with both fruits and vegetables in their diet showed a moderate reduction in upper respiratory tract infection (URTI) during pregnancy compared to women without or with fruit or vegetables food consumption (Li and Werler, 2010). It would seem that a potentially beneficial strategy is to eat more fruits and vegetables as part of a healthy and balanced diet.

Micronutrients are critical for the proper function of the immune system and play a vital role in promoting health and nutritional wellbeing when micronutrients are taken from a nutritionally balanced and diverse diet like fruits and vegetables and animal source foods. As per various epidemiological studies, the Mediterranean diet is a dietary type that is believed to be rich in balanced micronutrients and considered as one of the healthiest nutritional guidelines (Guasch-Ferre et al., 2017; Carlos et al., 2018).

### Dairy-Derived Supplements or Functional Foods

#### Bovine Colostrum

Bovine colostrum, also known as “early milk,” is the initial milk and is abundant in bioactive components produced by cows during the first 48 or 72h after birth. It is enriched in nutrients, both in terms of macronutrients and micronutrients compared to milk. The colostrum plays a vital role in improving body composition by building muscle mass and strength (Blair et al., 2020). Natural supplements help to lose body fat and muscle healing after strenuous exercise in athletes (Yoon and Kim, 2020). It has been shown that uptake of bovine colostrum (60g/day) for 8weeks with resistance training increases the leg press strength and decreases the bone resorption in older adults, male and female, compared to older adults taking whey protein supplements (Willoughby et al., 2018). A similar finding has been reported in physically active young males and females with bovine colostrum supplements (20g/day) for 8weeks (Główka et al., 2020). The decrease in body mass while taking colostrum could be attributed to the burning of fat. Additionally, colostrum is rich in calcium, and calcium is an essential factor in regulating lipid metabolism by reducing adipocyte lipid deposition and increasing lipolysis (Zhang et al., 2019).

Colostrum is abundant in immunoglobulins like IgG, IgA, and IgM that confer immunity (McGrath et al., 2016). In addition, bovine colostrum are a good source of other bioactive components such as growth factors like epidermal and platelet-derived growth factors, cytokines, vitamins like A, B, C, D, E, K, and antimicrobial factors antioxidant enzymes (McGrath et al., 2016). In recent years bovine colostrum has been studied in relation to nutrition and immunology for its role to confer immunity and good health. Recently, Davison (2021) in his review elaborated in detail about the immune properties of colostrum such as boosting immune functions or activating the immune cell by increasing neutrophil, oxidative burst and degranulation, as well as cytokine production by neutrophils and peripheral blood mononuclear cells. In addition, the low molecular weight fractions of bovine colostrum consumption supplement the phagocytic activity of monocyte and polymorphonuclear, along with momentary changes in the number of NK cells in gut mucosa (Jensen et al., 2012).

It is hypothesized that consumption of low molecular weight components of bovine colostrum elicits transient accumulation and release of new NK cells. Thus, the benefits of micro fractions of bovine colostrum in humans that are easily digested or pass through gut lining could be envisioned. However, such a hypothesis needs intensive investigation. Supplementation of bovine colostrum of 20g/day in male subjects for 4weeks acts as an immune booster in exercise-induced stress through significant increase in the neutrophil function and salivary lysozyme while lack of such effect has been observed after 12weeks of regimen (Jones et al., 2019). Of course, reduced salivary bacteria amount and incidence of upper respiratory illness during winter while doing intensive exercise have been documented. Even a low dose (3.2g/day) of bovine colostrum for 3days has been found to be effective in minimizing exercise-induced muscle damage (EIMD) without deteriorating the performance in soccer players (Kotsis et al., 2018).

Some studies also showed that after colostrum supplementation, secretary IgA concentration remained unchanged compared to the control group observed in IgA-deficient children through decreased infection severity or in athletes (Patiroglu and Kondolot, 2013; Shing et al., 2013). It was observed that individuals taking vaccination of oral *Salmonella typhi* Ty21a along with bovine colostrum for 1week showed an increasing pattern of IgA compared to controls though non-significant (He et al., 2001). Thus, as mentioned earlier, the results reflect and produce evidence favoring bovine colostrum beneficial effects by boosting immunity and rendering resistance to infection to normal healthy subjects or athletes doing intensive exercise.

In summary, the available evidence to date suggests that the benefits of consuming bovine colostrum are manifold. It benefits not only immune-compromised old adults or exercise-induced stressful athletes but also active humans. Research reports show the bovine colostrum supplementation manifests its beneficial effects just after 1h of consumption to 12weeks regularly. However, it seems that the duration of supplementation impacts the different types of immune functions. Being easily digestible and nutrient-rich has multiple functions, such as eliciting a systemic immune response in the gastrointestinal part and reducing upper respiratory tract infections. The duration and dose of the bovine colostrum need to be standardized to increase its efficacy for overall health benefits.

#### Probiotics

Probiotics are living microorganisms favorable to humans and are present in certain food items or provided as food supplements that reach the intestine in sufficient numbers to benefit the host’s health. It is well known that around 100 trillion micro-organisms harboring in the human gut that coevolved prevent pathogenic bacteria from transmitting through the gut, or else the imbalance cause diseases that are known during the time of Hippocrates (460–370 EC), who alleged that “all diseases begin in the gut” (Terpou et al., 2019).

The role of diet and other environmental factors modulating the density and metabolic activity of the human gut microbiota is recognized. Many beneficial bacteria are transported to gut *via* fermented foods such as fermented milk or dairy products (curd). Therefore the microorganisms, which are helpful for human health, have been commercialized in the form of probiotics. Initially, it was considered that probiotics only improve gut health; nonetheless, ample evidence supports the role of probiotics in conferring many aspects of immunity, specifically adaptive immunity (Azad et al., 2018). An adequate amount of probiotics positively modulates immunity by enhancing the intestinal mucosa and epithelial health’s pathogen blocking or barrier functions (Monteagudo-Mera et al., 2019). Specifically, probiotics proved helpful to people with poor immune systems suffering from frequent gastrointestinal infections caused by enteric bacteria, *Staphylococcus* and antibiotic-associated imbalance in gut microflora (Kothari et al., 2019; Stavropoulou and Bezirtzoglou, 2020). It has been reported that the gut microbial flora interacts with intestinal mucosal epithelial cells and activates the T17 helper cells (T_H_17) and regulatory T cells (T_reg_) that are involved in inflammatory response against helminths (Pandiyan et al., 2019).

The commercial probiotics also contain other strains of beneficial bacteria such as *lactobacillus*, *Bifidobacterium*, *Propionibacterium*, *Faecalibacterium*, and specific yeast species (Fijan, 2014). However, only certain strains are efficient and valuable to provide immunity and effectively combat infection when appropriately consumed. In addition, the immunity varies depending upon an individual’s age, gender, genetic constitution, and health status. Therefore the standard for the formulation, dose, and microbial strain type of probiotics should be considered as stated prior to treatment.

Several reports documented a negative impact on health as an outcome of the interaction between probiotics and host gut microbes, specifically in immune-compromised risk populations. The various adverse effects of probiotics are seen in risk groups such as people suffering from bacterial or fungal sepsis, systemic and localized infection, allergies, transfer of antibiotic resistance genes and bowel ischemia (Sanders et al., 2010; Zhang et al., 2015b). Majority of the adverse effects referred are associated with the *Lactobacillus*, *Bifidobacterium*, and *Endocarditis* species in probiotics. Besides, probiotic strains can also display antagonistic effects towards the host gut microbes like nutrient competition, co-aggregation with pathogens, and elicit response (De Melo Pereira et al., 2018).

Probiotics are also helpful in reducing upper respiratory tract infection (URTI) incidence and duration, though the reports are meager. A double-blinded, randomized controlled study has shown that if probiotics with specific bacterial composition are administered for 12weeks, it significantly reduces the URTI and cold symptoms in the probiotic group compared to placebo with a significant increase in immune components such as IFN-γ and sIgA in serum (Zhang et al., 2018). A decrease in duration of RTI symptoms and severity of symptoms with an increase in immune cells like T helper cells (CD4^+^) and T suppressor cells (CD8^+^) has also been noted after probiotics administration (Kim et al., 2018).

Probiotics have been observed to augment the serum cytokines, the efficiency of influenza vaccine and minimizing occurrence and duration of respiratory infections in COVID patients, so probiotics as an adjuvant to treat the COVID patients have been advocated (Darbandi et al., 2021). The reports regarding the effects of probiotics on the occurrence, duration, and severity of viral respiratory infections are inconsistent. In healthy adults, consuming a probiotic of specific strains followed by influenza virus vaccination resulted in a significant decrease in the frequency of antigen-specific IgA and T-helper type 1 response (Vitetta et al., 2017). The research reports regarding the response of probiotics to the vaccination are mostly in healthy adults or different probiotic strains. Therefore, randomized and placebo-controlled adjusted for age, vaccination, and composition of probiotics and administration duration may come out with overall benefits of probiotics (Maidens et al., 2013; Lehtoranta et al., 2014).

The side effect of antibiotics can also be reduced through probiotics following treatment. A large meta-analysis on 2,972 patients in intensive care units suffering from ventilator-associated pneumonia (VAP) showed a decrease in infection rate and incidence of VAP, suggesting that probiotics treatment is a promising way to treat patients (Manzanares et al., 2016). Nonetheless, more trials are recommended with different groups of patients to avoid side effects and increase the efficacy of probiotics. The reports from studies in animal models infected with influenza virus and then oral or mucosal administration of different strains of probiotics bacteria *Lactobacillus* also support and confirm earlier findings of using probiotics as a nutritional supplement or immune booster (Aponte et al., 2020).

Recent evidence and ongoing research suggest that the intestinal microbiota have a bidirectional effect on mood and psychiatric disorders. A meta-analysis of existing research reports showed administration of probiotics reduces depression, and it depends upon the dose and microbiota strain of the probiotics (Ng et al., 2018). Probiotics also treat inflammatory bowel disease and enhance cognitive functions through the gut-brain axis (Wasilewski et al., 2015; Bagga et al., 2018; Bermudez-Humaran et al., 2019; Zagorska et al., 2020).

It has been observed that the physically active people like athletes’ guts are rich in microbial population compared to those of sedentary people evaluated through fecal content (Clarke et al., 2014). Probiotics have been considered as important ergonomic supplements for improving the physical performance in athletes on short- or long-term consumption that is 2weeks to 3months besides, significant difference has been observed in the different diet and physical activity level between physically active and sedentary people (Marttinen et al., 2020). The gut microbiota influence an individual’s physical performance by modulating digestion and energy harvest during strenuous physical activity (Nay et al., 2019). A study in animals and humans found that probiotic microbe supplementation increased muscle mass, energy generation, and improved physical performance (such as Lactobacillus plantarum TWK10), or were able to modulate systemic and airway immune functions, such as serum TNF-level reduction immediately after marathon (like Lactobacillus casei Shirota; Chen et al., 2016; Vaisberg et al., 2019).

#### Prebiotics

The other way to modulate the gut microflora is through prebiotics; these are the undigestibale and unabsorbed food ingredients (mostly the oligosaccharides) that help to harbour useful bacteria population like Bacteroidetes and Firmicutes, and their activity for the host gut health (Vieira et al., 2013). Intake of fiberous food acts as prebiotics in the gut and modulates the mucosal immune functions and reduces the risk of enteric inflammation and like by increasing anti-inflammatory cytokines and reducing proinflammatory cytokines and the systemic immune response (Shokryazdan et al., 2017). The undigested food can also concert to short-chain fatty acids like butyrate that is absorbed by the gut and helps to reduce inflammatory disorders through increasing of T-regulatory cells (Treg) and reducing IFN-γ.

## Plant-Derived and Herbal Supplements

### Echinacea

Echinacea is the common name for a genus of plant species native to North America, also known as coneflower. *Echinacea purpurea* (purple coneflower) is most commonly used as herbal medicine to treat respiratory infections and in immune intervention studies. Echinacea has been observed to minimize the common cold incidence by around 58% and lessen the duration of infection by about 1.4days (Shah et al., 2007). The administration of commercial-grade of *Echinacea purpurea* supplement for 28days has been observed to improve the immune functions in the mucosal area after strenuous exercise in three consecutive 30s Wingate cycling tests by reducing the magnitude of exercise-induced decrease in salivary IgA concentration and release (Hall et al., 2007).

Hall et al. (2007) suggested that the possible immune function mechanism could increase cytokine production like IL-6 or 1, tumor necrosis factor, and activation of phagocytes and lymphocytes. *In vitro* cultures also reported that fresh *Echinacea purpurea* juice activates macrophages to produce cytokine (Lim, 2014). Several bioactive components are obtained from different parts of Echinacea, such as glycoproteins, caffeic acid phenolic compounds, flavanoids responsible for conferring both innate and adaptive immunity. A recent review elaborated on Echinacea’s immunomodulatory functions, such as inhibiting CD4^+^ and CD8^+^ T lymphocytes and improving macrophage phagocytosis by increasing lysosomal activity and nitric oxide production, re-establishing splenic NK cell activity, activating Th1 and Th2 cytokines for antibody production (Nagoor Meeran et al., 2021). However, a meta-analysis by Karsch-Völk et al. (2014) found that many Echinacea products slightly reduce the risk of catching a cold by 10–20% in healthy individuals.

The effectiveness depends upon the species, plant parts and bioactive content. In this line, Wang and Frueh, also did not notice any adequate treatment of common cold using Echinacea extract (Wang and Frueh, 2015). Echinacea has been shown in numerous studies to modulate both systemic and local immunity. The mechanisms immunomodulatory role of Echinacea supplements is not known currently. Nevertheless, it seems that the benefits come from bioactive compounds such as caffeic acids and derivatives, phenolic compounds, flavonoids, and alkalines (Sellami et al., 2018).

## Polyphenols and Other Plant-Derived Substances

Polyphenols are the compound that act as antioxidants and are present in medicinal plants as secondary metabolites, fruits, nuts, seed, vegetables, spices, cereals, and beverages. Polyphenol protects from harmful ultraviolet radiation and invading pathogens (Tungmunnithum et al., 2018). Flavonoids having various types (flavonols, flavones, and flavanones) show similar biological activities. Flavones include apigenin, baicalein, luteolin, and rutin. Quercetin and kaempferol are examples of flavonols. Hesperidin and naringin are two flavanones with growth-inhibitory effects in cancers such as colon, prostate, liver, stomach, cervix, pancreas, breast, and leukemia (Kumar and Pandey, 2013; Zhou et al., 2016).

Polyphenol consumption in diets is also reported to prevent the risk of developing a chronic and fatal disease like cardiovascular disorder, cancer, atherosclerosis, diabetes, osteoporosis and neurodegenerative diseases (Kozłowska and Szostak-Węgierek, 2017; Behl et al., 2020). Polyphenols like flavanoids or quercetin effectively cure cardiovascular disorders due to their antioxidant, anti-platelet, anti-inflammatory properties, augmenting endothelial or blocking expression of metalloproteinase 1 (MMP1) functions or chronic diseases cancer (Li et al., 2016). Polyphenols with immunomodulatory functions, such as aglycones epicatechin and catechin extracted from almond, demonstrated antimicrobial and antiviral activities against *S. aureus* and reduced herpes simplex virus titer *in vitro* (Musarra-Pizzo et al., 2020). Recently the oral administration of polyphenols to patients suffering from Nickel-Mediated Allergic Contact Dermatitis showed altered decease inflammatory biomarkers like interferon-γ, IL-4, IL-17, pentraxin three and NO. At the same time, the placebo group did not exhibit any change (Magrone et al., 2019).

The importance of polyphenols in URTI has also been studied. The incidence of exercise-induced URTI in cyclists has been minimized within 2weeks by taking 1,000mg of quercetin daily for 3weeks before and 2weeks after 3days (Myburgh, 2014). The daily consumption of nonalcoholic beer containing polyphenol in a fix quantity 1–1.5L/day for 3 wk. before and 2weeks after a marathon decreases the URTI incidence and IL-6 and blood leucocytes (Scherr et al., 2012). On the other hand, food items like dark chocolate having polyphenol eaten for 2weeks did not exhibit any significant immune effect measured through serum cytokine and cortisol despite a decrease in some oxidative stress markers (Stellingwerff et al., 2014). In conclusion, reports substantiated the benefits of polyphenols in some cases of providing immunity against the risk of URTI and combating infection. Nonetheless, the mechanisms behind this immunomodulatory role are still unclear, and further research could elucidate the mechanisms and pathways of the role of polyphenols in immune functions.

In summary, evidence is emerging for a potential beneficial effect of polyphenols on URTI risk and infection clearance. However, the mechanisms remain to be elucidated and more research is required to determine whether polyphenols should be recommended for immune support.

## Effects of Exercise and Diet/Nutrition on Immunity

Evidence shows that some benefits, particularly physical activity and a proper diet, can reinforce the immune mechanisms and prevent the inflammaging process (Weyh et al., 2020). It has been demonstrated that active exercise ameliorates the overall immune response in regularly trained people, conferring a wide range of advantages. As shown by the pioneering work of Nieman and Wentz (2019) women recruited for their regular practical exercises exhibited higher NK and T lymphocyte function compared to sedentary women of the same age who underwent 12weeks of exercise.

Among the beneficial effects of physical activity, lowering of CD14^+^ and CD16^+^ monocytes can also be counted, representing vital proinflammatory cells involved in the onset of immune diseases, such as rheumatoid arthritis and cardiovascular disorders (Ma et al., 2019; Mannucci et al., 2021). The role of exercise in regulating the expression of TLR (full form) was examined in individuals who underwent intense physical activity. TLRs are highly expressed on the cell surface of immune cells such as monocytes and macrophages. These receptors play an essential role in innate immunity and adaptive immunity, stimulating antimicrobial activity by activating the nuclear factor-kappa B (NFkB) pathway and releasing proinflammatory cytokines (Vijay, 2018).

As shown by Oliveira and Gleeson (2010) sustained cycling activity was able to reduce the TLR4 expression significantly by 45% after 1h of intense training in comparison with the control group. However, this effect was not permanent. The downregulation of TLR after prolonged exercise is not fully understood yet. Still, it may be caused by high levels of IL-6 released after training, which may compete with the physiological NFkB pathway of TLR, as discussed in several studies (Favere et al., 2021). Moreover, IL-6 secretion exhibits proinflammatory properties by stimulating the release of immunity regulators, including IL-10 and IL-1 receptor agonist (Dinarello, 2018).

According to the study of Spielmann et al. (2011) aerobic activity influences the frequency of T cells, leading to a decrease in senescent CD4^+^, CD8^+^, and CD28-CD57 T cells. In contrast, it causes a rise in the number of naïve CD8^+^ T cells. The reduced amount of senescent T cells can be explained through the mechanism of cell death, such as apoptosis, induced by intense physical activity (Minuzzi et al., 2018).

Although moderately exercising people experience less mortality and lower risk of infections than sedentary individuals, evidence also shows a higher tendency to suffer from upper respiratory tract infections (URTI) during intense or prolonged exercise in athletes. This may be due to changes in inflammatory pathways following low physical activity, resulting in higher susceptibility to pathogens (Jones and Davison, 2019).

Another interesting approach to strengthening the immune system is provided by nutritional strategies, which should work in synergy with exercise to maintain immune response efficiency. Proper nutrition must respond to pathogen stimuli, such as during fever, and avoid chronic inflammation. Conversely, malnutrition negatively affects the immune functions with variable effects according to patient age and extent of nutritional deficiency.

A plethora of micronutrients have been studied for their multiple properties in the maintenance of the immune system. The role of zinc as a cofactor for many proteins has been widely studied. As indicated by Andreini et al. (2006) more than 2000 human proteins are zinc-bonded to exert their physiological functions. The importance of zinc as a modulator of the immune system is evidenced by the impairment of innate and adapted immunity in zinc deficiency. In detail, decreased amount of zinc causes lower cytotoxicity of NK cells, reduced phagocytosis capacity of neutrophils, auto-reactivity of T cells and higher apoptosis rate of B cells (Maywald et al., 2017).

Glutamine is a pivotal modulator of the immune system, as observed through its ability to enhance the phagocytosis activity in macrophages, lymphocyte proliferation and cytotoxic activity in neutrophils. Glutamine is rapidly consumed by immune cells, especially during diseases, reaching a similar metabolic rate as glucose. Therefore, glutamine is generally used in the clinic routine for dietetic supplementations of immune depress patients (Cruzat et al., 2018).

Vitamin D is also thought to play an important role in modulating the immunity system, as confirmed by higher inflammation and increased risk of diabetes and rheumatoid arthritis in Vitamin D-deficient patients. Vitamin D exerts positive effects on innate immunity by increasing autophagy and chemotaxis in immune cells and reinforcing human physical barriers, such as intestinal and corneal tissues (Chirumbolo et al., 2017). Although vitamin D seems to stimulate regulatory T cells’ activity in many diseases, there are not enough data in the literature confirming a significant role of vitamin D in the regulation of adaptive immunity.

There is a strong link between gut microbiota and immunity because microbiota trains and stimulates immune system function, compensating for maintaining a symbiotic relationship with gut hosts. In addition, many studies have found that gut microbiota can help regulate immune homeostasis in innate and adaptive responses (Belkaid and Hand, 2014; Belkaid and Harrison, 2017).

According to the literature, exercise and diet modifications have a strong combined effect on immune system. This effect, however, can vary depending on the type, intensity and duration of the interventions. As shown by Roberts et al. (2006) the combination of daily aerobic exercise and a low-fat, high fiber diet in healthy patients and patients with metabolic syndrome induced a reduction of inflammation, leucocytes adhesion and chemotaxis capacity, and oxygen species production as well as a decrease in metabolic syndrome markers after 3weeks of treatment. Moreover, the experiments by Hill et al. (2007) suggested combining diet and exercising interventions to enforce the immunity and to prevent obesity, cardiovascular diseases and diabetes mellitus. Indeed, supplementation for 12weeks with fish-oil and moderate physical activity in overweight and obese volunteers was able to decrease the oxygen species production and maintain the bactericidal activity of neutrophils.

Additionally, Meksawan et al. (2004) demonstrated that different dietary fat intakes, associated with a short and intense exercise, gave variable effects in controlling pro-inflammatory markers after 3weeks of interventions in untrained healthy patients. Physical activity and proper nutrition or diet habits affect the process of immunosenescence and inflammation (Weyh et al., 2020), and boost the immunity that in turn improve the quality of life (Davison et al., 2016). In older adults, exercise and dietary protein intervention have shown to prevent muscles loss, improved muscle functions and quality of life (Strasser et al., 2018). Kommers et al. (2019) examined a positive association of probiotic supplementation on quality of life of constipated young female, while no impact of intense exercise on quality of life. Though there is meager report on the commercial immune booster supplements and active physical exercise in relation to improve or maintain a healthy lifespan, the nutritional supplements like single or MVM, dairy derived supplements, probiotics; all indeed act as aid to boost immune functions that is augmented by physical performances and in combination, so both in a combined way seem to maintain healthy life (Table 1).

**Table 1:** Common nutritional intervention and key food involved to stimulate human immune system.

Nutritional intervention and immunity	
Carbohydrate intervention	Restore exercise-induced immunodepression (Gunzer et al., 2012; Kerksick et al., 2018), role in endurance-exercise-induced adaption like mitochondrial biogenesis and lipolysis (Mata et al., 2019). Immunomodulatory effects like decrease in systemic IL-6 release neutrophils, monocytes, lymphocytes, reduce suppression of CD4^+^ and CD8^+^ T cells, causes leukocyte distribution, neutrophil degranulation and oxidative stress, lymphocyte proliferation and functions (Maydych et al., 2017; Peake et al., 2017). Increased-natural killer (NK; Gunzer et al., 2012; Clark and Mach, 2016). Minimized exercise-induced increased circulating cytokines, redistribution of immune cells like neutrophils, monocytes, NK cells and lymphocytes (Nieman and Wentz, 2019). Upper respiratory tract infections (URTI) recovery (Nieman and Mitmesser, 2017); reduction in risk of infection and mortality from viral infections like herpes simplex virus 1 [HSV-1] (Harper et al., 2021).
Micronutrients including (antioxidant vitamins-vitamins C and E); vitamin D and multivitamin	High doses of antioxidant vitamins (i.e., 10× RDA, recommended dietary allowance) effectively minimized the chances of infection in ultra-endurance athletes (Salehi et al., 2018); high dose of antioxidant decreased risk of URTI (Davison et al., 2016). Vitamins C and E in combination reduce oxidative stress in athletes (De Oliveira et al., 2019) Vitamin C inversely related to phagocytosis (Fantacone et al., 2020) Vitamin E before exercise under hypoxic condition at high altitude restores interleukin (IL)-6, TNF-α, IL-1ra, and IL-10 to normal level (Santos et al., 2016); decreases systemic inflammatory biomarkers in elite endurance athletes at altitude (Koivisto et al., 2019); in high dose acts as modulator proinflammatory agents, oxidative stress and NF-κB pathway having role in neuroprotection and cancer (Ungurianu et al., 2021). An association exists between vitamin D content and Acute Respiratory Tract Infection risk (Pham et al., 2019). Vitamin D participates in innate antimicrobial response through activation of Toll like receptors (TLR) that increased expression of both the 1-α-hydroxylase and vitamin D receptors (VDR; Chun et al., 2014). Vitamin D decreases reactive oxygen species production Neutrophil function decreased substantially in individuals with diet deficit in multiple vitamins and mineral lacks (Maggini et al., 2018). MVM supplements for at least 12weeks reduce the incidence of infection, increased vitamin C and zinc content (Fantacone et al., 2020) Zinc improves immunity of patients with sepsis (Alker and Haase, 2018)
Bovine colostrum	Improves body composition increase, muscle mass and strength and burn fat (Davison, 2021) Confer immunity; abundant in immunoglobulins like IgG, IgA and IgM that (McGrath et al., 2016). Good source of other bioactive components such as growth factors like epidermal and platelet-derived growth factors, cytokines, vitamins like A, B, C, D, E, K and antimicrobial factors like antioxidant enzymes (McGrath et al., 2016). Boost immune functions or activating the immune cell by increasing neutrophil, oxidative burst and degranulation, as well as cytokine production by neutrophils and peripheral blood mononuclear cells (Davison, 2021).
Probiotics	Enhances pathogen (enteric bacteria and staphylococcus) blocking or barrier functions of intestinal mucosa, epithelial health; confer adaptive Immunity (Georgieva et al., 2015; Terpou et al., 2019; Stavropoulou and Bezirtzoglou, 2020). In risk groups leads to bacterial or fungal sepsis, systemic and localized infection, allergies, transfer of antibiotic resistance genes and bowel ischemia (Kothari et al., 2019). Some strains show antagonistic effects towards the host gut microbes like nutrient competition, co-aggregation with pathogens, and elicit response (De Melo Pereira et al., 2018). Reduce URTI and cold symptoms with a significant increase in IFN-γ and sIgA in serum (Zhang et al., 2018). Increase serum cytokines, efficiency of influenza vaccine and minimizing occurrence and duration of respiratory infections in COVID patients (Darbandi et al., 2021). Reduce infection rate and incidence of ventilator-associated pneumonia (VAP; Manzanares et al., 2016).
Plant-derived and herbal supplements (including Echinacea, polyphenols and other plant-derived substances)	Provide both innate and adaptive immunity through its bioactive components (glycoproteins caffeic acid phenolic compounds, flavanoids; Jain and Pasare, 2017) Have immunomodulatory functions like inhibiting CD4^+^ and CD8^+^ T lymphocytes and enhancing the phagocytosis ability of macrophages by increasing the lysosomal activity and nitric oxide production, re-establish splenic NK cells activity, activating Th1 and Th2 cytokines for antibody production (Abel et al., 2018) Possess antioxidant and antibacterial properties, growth-inhibitory effects in different cancers like colon, prostate, liver, stomach, cervix, pancreas, breast, and leukemia (Zhou et al., 2016; Sharifi-Rad et al., 2018) Prevent the risk of disease like cardio-vascular disorder, cancer, arthrosclerosis, diabetes, osteoporosis and neurodegenerative diseases (Kozłowska and Szostak-Węgierek, 2017) Modulate gut immune system by increasing intraepithelial T cells and mucosal eosinophils, and activating inflammatory response against helminthes parasites (Motran et al., 2018) Altered decease inflammatory biomarkers like interferon-γ, IL-4, IL-17, pentraxin 3, and NO in patients of Nickel-Mediated Allergic Contact Dermatitis (Magrone et al., 2019)

## Nutritional Interventions in Ageing and Immunosenescence

Previous research has shown that a decrease in the number of peripheral blood naive cells, with a relative increase in the frequency of memory cells, is an irreversible age-related immune alteration (Quinn et al., 2018; Li et al., 2019). These two changes, along with inflammaging, are considered as the hallmarks of immunosenescence (decreasing strength of the immune system with age). However, improvements in health quality, immunization, and healthy and nutritious diets have increased the average lifespans of most developed-country citizens (Quilici et al., 2015). Immunosenescence is a complex process that affects the immune system as a whole and impairs the ability to respond adequately to invading pathogens.

The changes to the immune system seen with aging may not be permanent, and there is evidence of nutritional interventions promoting beneficial changes in immune cells (Yaqoob, 2017). Although there is enormous heterogeneity among individuals, mainly due to inherited differences in immune response genes that cannot be modified, certain factors, including lifestyle choices and nutritional habits, are flexible and significantly impact the progression of immunosenescence.

Nutrition has been studied as a modifiable factor in immune health for several decades, and this research has evolved into a field known as nutritional immunology. People in Mediterranean countries who consume many fruits, vegetables, legumes, unrefined cereals, skimmed dairy products, fish, and olive oil have a lower risk of heart disease (Caprara, 2018). Further, it is well established that nutrition directly impacts immunosenescence because deficiencies in several vitamins and minerals, protein-energy malnutrition, and excessive consumption of saturated fatty acids can hinder immune systems. Many studies have been conducted to investigate the effect of nutritional factors on the immune response. Thus, we discuss the role of macronutrients, multivitamins, minerals, and other nutrient components in the immunosenescence process.

To date, most nutritional interventions have concentrated on the use of dietary lipids; among them, micronutrients like vitamin E, carotenoids, and zinc are necessary for increasing immune responses. Vitamin E is the most important lipid-soluble antioxidant in the innate immune system of the cell. Dietary supplements of vitamin E have been shown to improve cell-mediated and humoral immune responses in many species. Several animal and human studies have found that supplementing with vitamin E increases lymphocyte proliferation, immunoglobulin levels, antibody responses, NK cell activity, interleukin (IL)-2 production and decreased IL-6 production (Mahalingam et al., 2011; Capo et al., 2016; Wang et al., 2017; Dalia et al., 2018). Evidence-based studies and clinical trials strongly suggest that vitamin E is effective in improving immune response. Especially significant improvement in Delayed-Type Hypersensitivity (DTH) and lowering the risk of infection in the people with >60years, and a daily dose of 200IU/d is most effective in improving immune function in the older people (Meydani et al., 2018).

Zinc is an essential mineral with its deficiency leading to reduced immune cell proliferation, cytokine production, and specific NK cell and neutrophil function reductions. The overall frequency of zinc deficiency worldwide is estimated to be higher than 20% (Wessells and Brown, 2012). Zinc can also have the ability to modulate the development of T helper cells, thereby reducing the cytokine storm, which is characterized by a sudden increase in circulating levels of different proinflammatory cytokines and chemokines. These are associated with a wide variety of infectious and noninfectious diseases and finally lead to systemic immune response impairment, resulting in acute respiratory distress syndrome (ARDS) or multiple organ failure (Griffith et al., 2014; Sokol and Luster, 2015; Soriani et al., 2018). Many studies indicate that age-related macular degeneration (AMD) is a progressive, degenerative disorder that commonly affects the elderly population and can lead to blindness. Further, it is treatable with zinc supplementation (Vishwanathan et al., 2013; Blasiak et al., 2020).

Every stage of immune response is dependent on the availability of specific micronutrients. A plethora of micronutrients have been studied for their multiple properties in the maintenance of the immune system. The importance of micronutrients in the immune system and infection was first discovered in vitamin C deficiency. In 1753, the first recorded controlled clinical trial was published (Noble et al., 2013). Although the recommended dietary allowances for older people indicate that their energy needs are lower than younger people’s, the micronutrient requirements are mostly the same. However, micronutrient deficiencies are more common in older people (Conzade et al., 2017). Lower food intake has been linked to less intake of calcium, iron, zinc, B vitamins, and vitamin E in older people, directly related to micronutrient deficiency (Fostinelli et al., 2020).

Fatty acids are an essential energy source, and they can influence immune cell functions by acting as precursors in the synthesis of lipid compounds. Fatty acid metabolic derivatives, such as eicosapentaenoic acid (EPA) and docosahexaenoic acid (DHA), are precursors for anti-inflammatory molecules that support monocyte recruitment in sites of infection (Schieffer et al., 2014). Fatty acids can have a dual effect on inflammation regulation. *In vitro* and *in vivo* studies show that the intracellular macromolecular complex Nod-like receptor protein 3 (NLRP3) inflammasome promotes proinflammatory cytokines such as IL-1 and IL-18. Additionally, metabolic derivatives of omega-3 fatty acids have been shown to prevent T cell differentiation into proinflammatory and decrease proinflammatory cytokine secretion (Chiurchiu et al., 2016).

Overall it appears that elderly individuals are more sensitive to the immunologic effects of fatty acid, which is advantageous and some disadvantages have also been observed with higher doses. As the doses used and the study designs differ between studies, more research is required to confirm the effects of fatty acid in this age group.

## Conclusion and Future Perspectives

The evidence suggests insufficient data to draw definite conclusions about which diets can boost the immune system prominently. Although the extent of the effects of resistance exercise on immune function remains unclear, most data show that lifestyle factors such as specific dietary preferences, physical activity, and nutrition influence the inflammatory profile (risk of infection). It is further associated with behavioral intervention to improve immune and overall health outcomes in subjects of all ages cooperatively and independently. On the other hand, it is understood that the global burden of chronic diseases is rising exponentially, necessitating long-term systemic strategies to increase daily physical activity levels. However, we know that exercise is a highly effective strategy for promoting a healthy immune system and lowering the risk of maladaptive immune aging. In this perspective, regular physical activity appears to be the most promising approach to combating cellular immunosenescence and inflamm-aging.

In addition, specific foods and supplements may also boost immunity and consequently reduce the risk of respiratory tract infection. Some emerging evidence suggests that other supplements such as bovine colostrum, probiotics, Echinacea, and polyphenols can also enormously boost the immune system. Beside, this study suggests that adequate intake of all good essential variety of fruits and vegetables can overcome the single nutrient deficiency.

In parallel, healthcare professionals and public health policymakers should encourage physical activity for everyone, but especially for older adults and other at-risk individuals, such as those suffering from chronic diseases related to aging and lifestyle. Thus, more short-term and long-term controlled, randomized interventional clinical trials using different exercise regimens with nutritional habits are needed to identify additional moderating factors and understand the mechanisms of immune cell mobilization and resistance to infections to guide clinical practice. This strategy would allow for a more personalized approach to treatment, which could help to improve the efficacy and acceptability of currently available therapies.

In summary, both exercise and a well-balanced diet can enhance immunity and resistance to infection in a large number of people. Immunosenescence is a new challenge for an aging population (those over 65years old), so interventions to boost immunity are incredibly beneficial for them. It has a beneficial effect on several immune functions. PA and following a balanced diet throughout life can help to reduce the adverse impact of age-related immune dysfunction. Although the evidence is still preliminary, we hope that this review will be helpful for researchers, clinicians, and academicians better understand the relationship between physical activity, nutrition, and immunosenescence.

## Author Contributions

TS, HV, BP, VC, WY, YC, and BL contributed substantially to the paper. TS, HV, YC, and BL contributed to conception and design of manuscript. HV, BP, and VC performed the methodological search on the research topic and helped write the draft manuscript. WY, YC, and BL critically revised the manuscript. All authors contributed to the article and approved the submitted version.

## Conflict of Interest

The authors declare that the research was conducted in the absence of any commercial or financial relationships that could be construed as a potential conflict of interest.

## Publisher’s Note

All claims expressed in this article are solely those of the authors and do not necessarily represent those of their affiliated organizations, or those of the publisher, the editors and the reviewers. Any product that may be evaluated in this article, or claim that may be made by its manufacturer, is not guaranteed or endorsed by the publisher.
